# A Review on Immune-Inspired Node Fault Detection in Wireless Sensor Networks with a Focus on the Danger Theory

**DOI:** 10.3390/s23031166

**Published:** 2023-01-19

**Authors:** Dominik Widhalm, Karl M. Goeschka, Wolfgang Kastner

**Affiliations:** 1 Department Electronic Engineering, University of Applied Sciences Technikum Wien, 1200 Vienna, Austria; 2 Automation Systems Group, Faculty of Informatics, TU Wien, 1040 Vienna, Austria

**Keywords:** fault detection, node fault, immune mechanism, artificial immune system, sensor node, wireless sensor network

## Abstract

The use of fault detection and tolerance measures in wireless sensor networks is inevitable to ensure the reliability of the data sources. In this context, immune-inspired concepts offer suitable characteristics for developing lightweight fault detection systems, and previous works have shown promising results. In this article, we provide a literature review of immune-inspired fault detection approaches in sensor networks proposed in the last two decades. We discuss the unique properties of the human immune system and how the found approaches exploit them. With the information from the literature review extended with the findings of our previous works, we discuss the limitations of current approaches and consequent future research directions. We have found that immune-inspired techniques are well suited for lightweight fault detection, but there are still open questions concerning the effective and efficient use of those in sensor networks.

## 1. Introduction

Our information society is hungry for data. We gather and analyze an ever-increasing amount of data captured from an expanding number of sources. These data are essential for a plethora of data services that provide us with insights into existing processes or predictions for future events, both being used in industry and academia. Examples include process automation, precision agriculture, or research that leverages the available data for event detection, trend prediction, process analysis, or decision support. The data services heavily depend on the input data’s timely availability and fine-grained quality. Inaccurate or false data lead to erroneous information and can ultimately result in incorrect findings and/or wrong (counter-)actions.

In this context, wireless sensor network (WSNs) have become an essential source of fine-grained data about phenomena or events. Today, they are used in a wide range of services (cf. [1]). WSNs consist of wirelessly connected sensor nodes deployed in an area of interest to monitor physical quantities close to their source and, thus, provide data with a high level of detail. In most applications, the sensor nodes perform some (pre)processing of the measurements and forward the data to central services for further processing (i.e., cloud systems). In these central services, the data is eventually fed to statistical machine learning, or other methods as part of the data services to extract information.

However, during the data analysis, data instances may deviate from an expected or previously learned “normal” behavior. The sensor data can have outliers, show suspicious behavior in the form of offsets and/or drifts, or differ from the data reported by other sensor nodes in the same neighborhood. As such, anomalies can potentially reflect an event in the observed area, anomaly detection approaches are widely used to detect data events in WSN applications.

Nevertheless, not all anomalies are related to actual events in the monitored environment; they can also stem from faults in the data chain. Especially sensor node faults have been found to negatively impact the overall quality of the data reported by a WSN. Sensor nodes are dedicated embedded systems with strictly limited resources that often prevent the use of well-established fault-tolerance concepts, such as hardware and/or software redundancy. Most of all, the energy budget available on the sensor nodes is bounded since most nodes are battery-powered. They are expected to operate over long periods of time without the possibility of battery recharging or replacement. Additionally, sensor nodes usually consist of low-cost components that are prone to experiencing faults when being operated under unpredictable and uncontrollable conditions imposed by outdoor environments. Consequently, proper runtime measures are inevitable to ensure the correctness and accuracy of the data reported by the sensor nodes.

Over the years, numerous approaches and concepts that meet the requirements of sensor nodes have been proposed to tackle the problem of fault detection in WSNs. In this context, concepts inspired by the natural anomaly detection capabilities of the human immune system have gained broad interest from the research community. The immune system offers desirable properties for computing systems, such as its widely distributed and decentralized operation, its ability to perform temporal and spatial correlation, and its high detection rate with a low false alarm rate. Therefore, many researchers took inspiration from immune mechanisms when developing novel approaches for fault detection in resource-constrained systems such as WSNs.

In this article, we contribute with a review of immune-inspired fault detection approaches for WSNs proposed during the last two decades. Previous reviews are either outdated, focus on fault detection or immune-inspired techniques in separation, or target other areas of application than WSNs. Based on our review, we discuss the state of the art for immune-inspired detection approaches and highlight the beneficial properties of such mechanisms for fault detection on resource-limited sensor nodes. Nevertheless, the information from the literature review in combination with our findings from previous research (cf. [2]) revealed that current approaches entail several limitations and shortcomings. Consequently, we provide a discussion of these open problems and present future research directions necessary to cope with these.

### 1.1. Immune-Inspired Fault Detection

Sensor nodes are key components that significantly influence the sensor networks’ dependability, especially concerning the reliability of the data sources (cf. [3]). They need to operate in a reliable and energy-efficient way to ensure accurate data acquisition while operating unattended for long times. The prevalent use of low-cost components and the strictly limited resources in combination with the often harsh environmental conditions make sensor nodes prone to various types of faults. Therefore, fault detection or fault tolerance measures are crucial to ensure a reliable operation.

One branch of node fault detection schemes applicable to WSNs comprises immune-inspired approaches. Such approaches have shown promising capabilities and preliminary results for developing lightweight fault detection systems.

In [4], the authors claim that “*… the process for characterizing a sensor network fault or anomaly is very similar to diagnosing an illness*”. The authors of [4] draw the connection between the task of anomaly or fault detection in computing systems and the basic principles of immune systems. Similarly, the authors of [5,6] consider the normal behavior of a computer system to be free of anomalous occurrences, hence, “healthy”. Detecting unhealthy circumstances is precisely what the human immune system (HIS) achieves. In other words, anomaly detection systems and the HIS share the same goal, which is to keep the system stable despite a continuously changing environment [7]. Thus, applying immune-inspired techniques to detect deviations from a “normal” operation seems reasonable. Regarding the use in WSNs, the authors of [8] go even further and claim that the basic architecture of sensor networks has a high structural similarity to the biological cell structure.

For this reason, a new discipline arose in the early 1990s aiming at deriving computational models inspired by concepts from immunology, namely, the field of artificial immune systems (AISs) [9] (sometimes also referred to as computational immunology or immuno-computing). AISs are bio-inspired schemes leveraging the immune system’s characteristics for use in computational problem-solving [10,11]. The field of AISs encompasses a collection of algorithms that are models or abstractions of mechanisms observed in the HIS [12,13,14,15]. New insights into the functioning of the HIS and the processes involved in detecting infections and regulating the responses serve as inspiration for novel computational models.

### 1.2. Related Work

There are several reviews and surveys of immune-inspired approaches or fault detection techniques for WSNs available. Nevertheless, they are mostly outdated (published before 2010), do not consider WSNs or focus on either immune-inspired approaches (mainly for security applications), or fault diagnosis in separation. But we did not find any recent review that targets WSNs and includes immune-inspired fault detection approaches.

We found several older reviews for computer networks that utilize immune mechanisms for intrusion detection systems (IDSs) [13,16,17,18]. Their included approaches are neither (directly) applicable to WSNs nor do they consider fault detection. Additionally, as they were published before 2010, they do not include the latest findings and developments of immune-inspired computing.

Similarly, more recent reviews and surveys focus on either immune-inspired security applications, target other types of systems, or do not consider approaches for fault detection based on immune mechanisms. For example, the survey provided in [19] discusses immune-inspired anomaly detection in WSNs, but focuses on their application for network intrusion and attack detection only. The authors do not include immune approaches for fault detection or diagnosis.

On the other hand, the survey presented in [20] targets multi-robot systems that have different characteristics from WSNs. Although the goals of fault detection in both types of systems are similar, their specifics and requirements differ, and so do the approaches that are feasibly applicable in both areas.

Moreover, there are reviews of approaches for fault detection in WSNs published in recent years, such as the surveys in [21,22]. In both surveys, however, only techniques based on statistics, correlations, or simple self-checks are discussed, but they do not include immune-inspired approaches. In the same way, past reviews and surveys often did not consider immune-inspired approaches in their discussion (cf. [23]), although such techniques have been shown suitable for lightweight fault detection in WSNs.

Consequently, we provide a review of immune-inspired fault detection approaches in this article that targets WSNs in particular and covers the last two decades, including recent works in the field. In addition, we highlight current research gaps for effective and efficient fault detection revealed by the literature review and strengthened by our findings from previous works [2].

### 1.3. Article Outline

The remainder of this article is structured as follows. We first present a concise history of immunological theories in Section 2 followed by an elaboration on the unique properties of the immune system in Section 3. Next, we briefly introduce the danger theory in Section 4. The four classical AIS theories are then presented in Section 5. One of the most commonly used immune-inspired algorithms for fault detection is the dendritic cell algorithm (DCA), as discussed in Section 6. In Section 7, we provide our literature review of immune-inspired fault detection approaches applicable to WSNs, where we particularly consider concepts based on the danger theory (i.e., DCA-based approaches). Based on the findings of our review, we elaborate on the open problems of current approaches and possible research directions to solve them in Section 8. With Section 9, we conclude this article with a short recap of the main findings of our review on immune-inspired fault detection approaches tailored for WSNs.

## 2. History and Immunological Theories

Several works [24,25,26] define the beginning of immunology with the discovery of the basic principle of immunization and phagocytosis by Pasteur and Metchnikoff in 1870. In 1890, von Behring discovered the presence of antibodies in the body of mammals, followed by the detection of cell receptors by Ehrlich around 1900. Based on the work of von Behring and Ehrlich, Bordet and Landsteiner found in the 1930s that antibodies have a particular specificity. Thus, they only react to certain other types of cells. From there, it took another 20 years until La Verne and Burnet started to work on a theory on clonal selection of specific lymphocytes (B and T cells in particular) in the 1950s, on whose basis Burnet developed the clonal selection theory in 1957 (cf. [27]).

**Figure 1:**
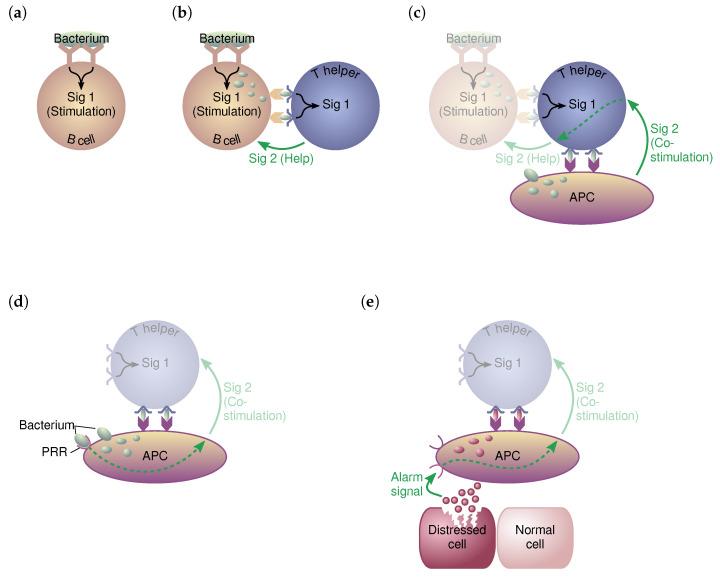
A history of immunological models (after [25], Figure 1). (**a**) SNS (1959); (**b**) Two-signal model (1969); (**c**) Extended two-signal model (1975); (**d**) INS (1989); (**e**) Danger theory (1994).

These fundamental discoveries led to the development of the so-called self/non-self (SNS) model (or “one-signal model”) in 1959 [28]. The name “self/non-self” refers to the basic process of B cells, which is distinguishes between entities that originate from their own system (“self”) and those that are foreign to the host (“non-self”). Similarly, the name “one-signal” model originates from the primary hypothesis that the immune reaction is triggered by recognizing non-self entities. As a result, only one signaling factor is required to trigger an immune response (see Figure 1a).

Soon, the SNS model was challenged by Oudin et al. [29] with questions that the original model could not answer. As a consequence, Bretscher and Cohn proposed in 1969 their associative recognition theory, sometimes referred to as the “two-signal model” [30]. In their model, antigen recognition alone is insufficient to trigger an immune response. It requires a second “signal”, which they named *help signal* as shown in Figure 1b. This help signal is necessary to trigger the B cells. If only Signal 1 (antigen recognition) is present without the secondary help signal, the B cell simply dies.

Meanwhile, Jerne was working on another aspect of the HIS, which he published in 1973 as his idiotypic network theory [31]. Jerne focused on the interaction of the particular parts of the immune system and suggested that the immune system consists of complementary idiotypes and paratopes that coexist and form some kind of a formal network. The idiotypes and paratopes act as stimulatory or suppressive factors in this network. Usually, these factors are balanced. In case the stimulatory parts become rife, an immune response is triggered. For a long time, the idiotypic network theory was seen as a competitive model to Cohn and Bretscher’s associative recognition theory. However, today, Jerne’s propositions on the regulation of the HIS by such an idiotypic network is considered complementary to the prevalent models of immune response activation [32].

Lafferty and Cunningham further refined and extended the two-signal model in 1975 [33]. As depicted in Figure 1c, they claimed that the T helper cells themselves need to be co-stimulated by antigen-presenting cells (APCs) (e.g., dendritic cells (DCs)) to provide the help signal to the B cells. If the T helper cell recognizes Signal 1 (antigen recognition) but receives no co-stimulation from an APC, it dies. Consequently, it does not relay the co-stimulation as a help signal to the B cell, causing this to die, too. Thus, the presence of two signaling factors in conjunction is needed to trigger an immune response by activating the B cells:

1. Antigen recognition (i.e., the affinity between T cell receptors and certain antigens);
2. Co-stimulation by T helper cells.

The extended two-signal model by Lafferty and Cunningham served as a sound basis for the functioning of the HIS and stayed untouched for quite some time. Later, new observations on how vaccines worked led to questions not answerable by the model. In particular, it was found that adjuvants were needed in combination with vaccines to stimulate immune responses. It was Janeway in 1989 who presented a new refined model of the immune system, the infectious non-self (INS) model (cf. [34]), as shown in Figure 1d. This model suggests that the APCs themselves need to be activated before being able to provide the co-stimulation signal. For this reason, the APCs have their own form of SNS discrimination that is based on the detection of conserved pathogen associated molecular patterns (PAMP) (essentially exogenous signals) through pattern recognition receptors (PRRs).

For a long time, it was believed that the critical element in activating immune responses is antigen recognition, that is, the discrimination of entities that originated from their own system (“self”) from those that are foreign (“non-self”). This “self/non-self” view became increasingly challenged by observations that the model could not explain, for example, transplants (no attack against “non-self”) as well as tumors or autoimmunity (both attacks of “self”). Another prominent example is the absence of immune responses to foreign bacteria in the gut or the food we eat [26]. As a consequence, the model of the HIS was continuously refined to be able to explain new findings. However, the core mechanisms remained the discrimination between self and non-self.

This view was significantly changed when Polly Matzinger presented her “danger theory” in 1994 (cf. [25,35]). According to this theory, the immune system reacts to entities causing damage rather than those considered foreign. Unlike the INS model, the danger theory builds upon the suggestion that DCs are natural information fusion entities able to combine cellular signals from both endogenous and exogenous sources [25,36]. The cellular signals are further distinguished based on their origin. As highlighted in Figure 1e, there are signals from distressed or injured cells (necrotic signals) that imply danger. In contrast, cellular signals from cells that died naturally (apoptotic signals) present a somewhat safe situation [7].

However, even today, immunologists are not fully sure how the immune system works in its entirety and which entities and processes are actually involved. So far, the INS model and the danger theory are two of the most hotly debated theories, and their basic principles are accepted by the majority of immunologists [37]. Still, the danger theory implies some problems such as those of previous immune models. Similar to the question of how to discriminate self from non-self of the SNS model [14], the danger theory faces the difficulty of how to distinguish between danger and non-danger [26].

## 3. Unique Properties of the Immune System

The human body is unquestionably one of the most complex systems known to humanity. There are three main regulation systems in the human body:

- The nervous system.
- The endocrine system.
- The immune system.

These three systems are integrated into one ultimate information communication network within the human body [38]. However, each regulation system has its specific roles and unique properties. Understanding these unique properties is necessary for building effective and efficient computational models based on mechanisms and processes observed in natural systems.

In the following, we will first provide a brief overview of these three regulation systems in Section 3.1. Then, the multi-layer defense mechanism of the HIS is presented in Section 3.2. Finally, the role of leukocytes and, in particular, the lymphocytes is discussed in Section 3.3.

### 3.1. Nervous, Endocrine-, and Immune System

The nervous system is a highly ramified network with a hierarchical order controlled by a central controller (the brain). Information is transported via electrical impulses that can be amplified or blocked by messengers. The nervous system, and particularly, the brain have been used as inspiration for computer scientists for a long time (e.g., in artificial neural networks (ANNs)).

On the other hand, the endocrine system is a regulation system purely based on chemical messengers (i.e., hormones; cf. [39]). These chemical messengers are secreted by different source organs (called glands) in the human body. The regulation itself happens with specific feedback loops of the hormones as almost every hormone has a complementary hormone [40]. The endocrine system tries to establish homeostasis (or feedback inhibition) between the chemical messengers by regulating the secretion of the respective complementary hormones. The endocrine system has some interesting properties [41], such as self-organization, synchronization, and cascading effects, that offer inspiration for certain computational problems.

In [41], Sinha and Chaczko compared the basic structure and working principle of the endocrine system with large-scale Internet of Things (IoT) infrastructures. Based on this view, they argue that models derived from the endocrine system offer great potential to solve problems prevalent in such large-scale networks. For this reason, several computational models based on the endocrine system have been proposed in the past, such as the autonomous decentralized system [42,43,44], the digital hormone system used for self-organized robot swarms [45,46,47], the computational model of hormones as first proposed in [48] and extended in [49], the regulation model of hormones [50,51], as well as the artificial hormone system [52,53].

The third regulation system, the immune system, is a widely distributed and inherently parallel network of a significant number of diverse entities. These entities work simultaneously and in cooperation with each other to reach the overall goal, to keep the body healthy [54,55]. It is a decentralized system without a central controlling instance (such as the brain for the nervous system). One of the most significant advantages of the HIS is its vast amount of resources. The immune system of an adult consists of around $10^{12}$ lymphocytes, $10^{20}$ soluble antibody molecules with about 5 million different antibody types, and a daily turn-over of these components of approximately 2% (cf. [24]).

Additionally, the HIS operates on different levels using various components, such as physical barriers (e.g., skin), chemical barriers (e.g., antimicrobial substances such as sweat and saliva), cellular proteins (e.g., cytokines), and a large number of different cells (e.g., macrophages and DCs). All these components and their interactions build up a highly complex self-organizing system with beneficial properties, such as error tolerance, adaptation, and self-monitoring [56]. Certain parts of the immune system even have learning, memory, and associative capabilities (cf. [57]).

### 3.2. Innate and Adaptive Immunity

The immune system has an ingenious multi-layer defense mechanism consisting of two distinct yet interrelated immune mechanisms [58]:

- Innate (non-specific) immunity.
- Adaptive (specific) immunity.

The combination and interaction of both form versatile and efficient protection for the human body. Both parts of the immune system use many different cells of diverse specialization to protect the host efficiently.

#### 3.2.1. Innate Immunity

The innate immune system [59] provides non-specific protection and defense mechanisms, as well as general immune responses. There are four types of defense barriers in innate immunity, namely (cf. [58]):

1. Anatomic barriers;
2. Physiologic barriers;
3. Endocytic and phagocytic barriers;
4. Inflammatory barriers.

Innate immunity consists of a large number of different cells providing a defense against the general properties of pathogens [60]. Hereby, the APCs (a kind of leukocyte, or more specifically monocyte) play an important role, especially the DCs (see Section 4.3). The innate immune system is an essential first line of defense against invading pathogens using generic responses [61]. The innate immune system does not develop memory and, thus, does not offer specific responses [62]. A review of innate immunity and its biological principles and properties can be found in [63].

#### 3.2.2. Adaptive Immunity

The adaptive immune system [64] provides more specific and compelling response mechanisms, as well as the capability to learn from previous occurrences of pathogens (i.e., immune memory [65]). It is sometimes called *acquired immunity* as the specific responses are developed over the lifetime of the host [56]. The main components of the adaptive immune system are lymphocytes, in particular B and T cells. In contrast to the leukocytes constituting the innate immunity, these cells can evolve over the lifetime of the host by specializing their receptors [66]. Based on these cells and their contribution to adaptive immunity, two primary adaptive immune responses can be distinguished, the *humoral response* and the *cellular response* [62,67,68].

The *humoral response*, or humoral immunity, refers to the interaction of B cells with antigens by producing specific antibodies that detect and eliminate foreign entities. B cells are produced by the bone marrow, where they have to survive a negative selection process before being released into the bloodstream. This negative selection process is part of the SNS theory and makes sure that the B cells surviving are self-tolerant; thus, they do not attack native (self) cells. If a B cell matches a particular antigen, it responds by multiplying itself through clonal expansion. In this process, B cells divide into several clones with slightly mutated antibodies to cover a broader spectrum of antigens and increase the chance of an even better antigen matching [69]. B cells with a high affinity can evolve to memory B cells capable of identifying the same pathogen much faster in the future (as the activation and stimulation process is shorter for memory B cells [70]). Such an immune response from memory B cells is called immune memory (also referred to as secondary immune response or strong immunity [65]) and provides an essential characteristic of the adaptive immune system, namely the ability to learn through interaction with the environment. Approximately 90% of the B cells die after their responses or lifespans, and the rest remain as memory cells [71].

The second adaptive immune response is the *cellular response*. It refers to the behavior of T cells that have two main tasks:

- The detection of intrusions by T helper cells (T_h_).
- The attraction of cytotoxic T cells (T_c_) for the disposal of infected cells [68].

To be more precise, the T_c_ becomes activated on the recognition of infected cells and starts producing molecules that destroy the infected cell.

In addition to T_h_ and T_c_, there exists a third type of T cells, the regulatory T cells. These regulatory T cells exist in two different stages: naive or active [71]. After being produced in the bone marrow, these regulatory T cells migrate to the thymus where they undergo a negative/positive selection process similar to B cells (but in the thymus instead of the bone marrow). Regulatory T cells that survived the selection process and that have not experienced an antigen yet are called *naive T cells*. Naive T cells can become *activated T cells* if they successfully bind to an antigen in combination with co-stimulation from an APC (or DC to be precise; see immune models in Section 2). Thereby, the degree of activation depends on the degree of signaling from the DC. In the case of excessive levels of co-stimulation, the T cells die to prevent overly excessive immune responses, a process called activation-induced cell death [72].

### 3.3. White Blood Cells

**Figure 2:**
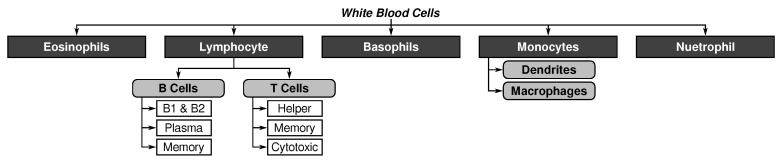
Classification of the white blood cells (adapted taken from [73], Figure 2).

Although many cells are involved in immunity, white blood cells build the core of the immune system. These cells are primarily produced and matured in lymphoid organs (e.g., thymus or bone marrow). As depicted in Figure 2, they are categorized in general white blood cells,  *leukocytes*, and specific subtypes of white blood cells,  *lymphocytes* [9].

While leukocytes form the basis of innate immunity (i.e., monocytes such as macrophages and APC), the adaptive immune responses are primarily performed by lymphocytes (i.e., T and B cells, as well as natural killer cells) [25]. The three most important white blood cells for immunity are:

- **dendritic cell (DCs)** are a particular class of APCs that moves in blood and processes information about antigens and dead cells found in their way.
- **T cells** are produced by the bone marrow and are responsible for destroying infectious cells.
- **B cells** are also produced by the bone marrow and stimulate the production of antibodies.

Due to their way of detecting foreign antigens, the antibodies are often called detectors, especially in the context of AIS.

In addition to the white blood cells, a large number of other cells and molecules are essential for the functioning of the immune system. Thereby, the ligands (or keys) play an important role as they are responsible for activating the cells’ receptors. As with the endocrine system, also the immune system contains regulating molecules called cytokines. Additionally, chemokines are specialized molecules that stimulate cell movement [9].

Altogether, the immune system shows characteristics also found in other bio-inspired systems. As the cells and their interaction share similar properties with swarm-like systems, the immune system is often considered a swarm system, too [74]. Also, to detect foreign entities, the immune system uses affinity measures that are, in their fundamental principle, similar to the fitness function in genetic algorithm (GA) [67]. A detailed overview of the (natural) immune system can be found in [58,75].

## 4. The Danger Theory

**Figure 3:**
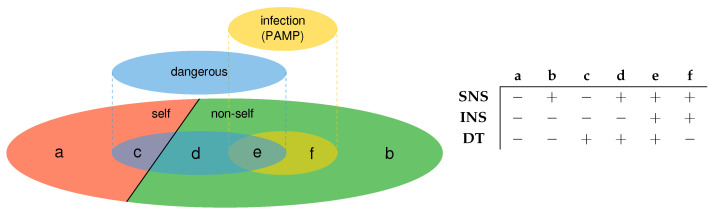
Antigen responses of different immune theories (after [25], Figure 2).

The danger theory states that the immune system does not primarily react to foreignness but to circumstances that pose a danger to the host. Therefore, it changes the discrimination of “self from non-self” of the SNS model to a discrimination of “some self from some non-self” depending on the presence of danger to the system (see Section 4.1). This difference in the antigen discrimination is shown in Figure 3, where SNS refers to the self/non-self model, INS to the infectious non-self model, and DT to the danger theory. In the figure, a “+” states that the theory reacts to this kind of antigens, while a “–” means that the theory ignores antigens of that kind.

In the danger theory, the danger is represented by the presence of so-called danger signals in the absence of down-regulating safe signals within a specific area (refer to Section 4.2). These necrotic (danger) signals and apoptotic (safe) signals in combination with PAMP are integrated by the dendritic cells to instruct the immune system to respond appropriately. Thus, the dendritic cells are major control mechanisms in immune systems (cf. Section 4.3).

### 4.1. Basic Concept

While previous immune models often focused on the role of adaptive immunity, Matzinger also stressed the importance of innate immunity [25,35]. From a biological point of view, the innate immune system has three main roles [57]:

- Defending the host in the early stages of infection.
- Initiating adaptive immune responses.
- Determining the actual type of adaptive response through APCs (i.e., DCs).

In the danger theory, signal two is provided by “professional” APCs, the DCs, which provide a vital link between innate and adaptive immunity [37]. Due to their way of collecting and evaluating the information on the current condition of the host, these DCs are sometimes denoted as the crime-scene investigators of the HIS [76]. Therefore, the danger theory suggests that there are two key elements responsible for immunity:

- The tissue with the signals contained.
- The alignment of innate and adaptive immunity by DCs.

The signals are discussed in more detail in Section 4.2.

As a result, the danger theory further implies a notable change regarding the control of immune responses. It highlights the role of the tissue for the immune system as it suggests that it is the tissue that controls immune responses and the evolution of the immune system [37,77].

The danger theory was initially hotly discussed within the immunology community and not accepted by all members [78]. However, Matzinger and other advocates of the danger theory found more and more evidence for their claims, as well as observations in nature that can not be explained by the previously prevalent theories. The danger theory states that the “foreignness” of a pathogen alone is not enough to trigger an immune response and that, on the other hand, “selfness” is no ultimate guarantee of tolerance [25]. As shown by Matzinger in [35,79], changes do happen in the human body over lifetime, be it of natural cause (e.g., pregnancy) or due to external intervention (e.g., surgeries); thus, the self changes as well. More detailed information on the danger theory from an immunologist’s view can be found in [25].

### 4.2. Immunological Signals

The danger theory states that the affinity between an antigen and an antibody (“signal one”) is not enough to trigger an immune response [58,80]. In addition, there needs to be a co-stimulation by APCs, such as the DCs (“signal two”; see Figure 1). DCs reside in the tissue and collect antigenic material and contextual information (commonly termed signals). According to the danger theory, it is the correlation of the contextual information (i.e., the signals) that triggers immune responses. Matzinger [35] groups these signals into three main categories (see also [66]):

- **Apoptosis**: natural death of cells (the “safe signals”).
- **Necrosis**: unnatural death of cells (the “danger signals”).
- **PAMP**: biological signatures of potential intrusions (e.g., foreign bacteria).

The danger signals can be further divided into endogenous (generated by the body, such as heat shock proteins, nucleotides, neuromediators, and cytokines) and exogenous (caused by invading organisms) [81]. These necrotic (danger) signals and apoptotic (safe) signals in combination with PAMP signals are integrated by the DCs to instruct the immune system to respond appropriately [66]. For more information on necrosis, apoptosis, and their processes and characteristics, we refer an interested reader to [82].

However, Matzinger admits that the exact nature of the danger signals is unclear, resulting in the difficulty of discriminating danger from non-danger [26]. Since the advent of the danger theory in 1994, many signals affecting the DCs have been empirically revealed [81]. As argued by Aickelin and Cayzer [26], a connection to the classical SNS theory is to consider the presence of non-self as a kind of danger signal.

### 4.3. The Role of Dendritic Cells

The danger theory focuses on the DCs since they can stimulate naive T cells, and thus, initiate primary immune responses [81]. DCs are monocytes (i.e., white blood cells) that were initially identified by Steinman and Cohn [83] and are native to the innate immune system [36]. Due to their function, they can be seen as the body’s own intrusion detection agents [84]. DCs provide a vital link between the innate and the adaptive immune system as they link the initial detection (innate) to the actual effector response (adaptive) [37]. Additionally, DCs are one of the major control mechanisms in immune systems as they coordinate the T cell responses by producing certain pro- or anti-inflammatory cytokines (chemical messengers). Pro-inflammatory cytokines have an activating effect on immune responses, while anti-inflammatory cytokines have a suppressing effect.

DCs are produced by the bone marrow and exist in three states of maturity with different functions, respectively [63,85]. After being produced, the DCs are in an *immature* state (denoted as iDC). The iDCs reside in the tissue and have the primary task of collecting cellular debris via ingestion [37,86]. Thereby, they collect antigens and receive the signals mentioned above (i.e., danger, safe, and PAMP). After being exposed to a certain quantity of signals, the iDC becomes activated. Exposure to PAMPs accelerates the process of maturation.

The activated iDC then migrates from the tissue to the lymph nodes where they either become *semi-mature* (smDC) or *mature* (mDC). In case the iDC experiences a higher concentration of danger-related signals (i.e., a greater quantity of either PAMP or danger signals), it maturates into an mDC. Otherwise, it becomes an smDC.

In the lymphoid tissues, the smDC and mDC interact with naive T and B cells to either initiate (in the case of mDC) or suppress (in the case of smDC) an adaptive immune response. The naive T cells respond by differentiating further into activated T cells (see Section 3.2.2, as well as [71]). This is achieved by the production of small quantities of anti-inflammatory cytokines by the smDC and the production of pro-inflammatory cytokines by the mDC, respectively. In addition, the mDC produces co-stimulatory molecules that have an amplifying effect on both the PAMPs and danger signals in the surrounding area [87].

However, also iDC have a suppressing effect as the encounter of iDC with T cells results in the deactivation of the T cell due to a lack of co-stimulatory molecules or inflammatory cytokines [86]. The DCs do not perform their function in isolation as there are numerous of these DCs in the tissue. Thus, they form a population-based system offering high error tolerance and robustness through diversity, as well as a low false alarm rate (FAR) [84]. Further information on the DCs functioning with a focus on AIS is available in [88].

### 4.4. Impact on Computational Problems

The advances in immunology research made over the last century not only help us to understand the working principle of the HIS and derive appropriate therapies in medicine, but they also provide a great source of inspiration for other scientific disciplines. In the area of computer science and engineering, findings on biological processes have often served as inspiration for novel techniques to solve computational problems (i.e., bio-inspired computing [89]).

Concerning fault detection techniques, especially the findings of Matzinger’s danger theory, have led to a paradigm shift on how to detect faults in a system. While past approaches mostly followed the negative/positive-selection approach to distinguish between normal and faulty system states, more recent approaches are increasingly inspired by the danger theory. In contrast to previous immune theories, the danger theory states that the immune system aims at identifying circumstances that pose a danger to the host rather than identifying everything foreign. In doing so, the HIS involves a multitude of diverse immune entities (i.e., cells) to form a self-organized, distributed, and cooperative defense mechanism where the single cells perform merely simple tasks.

As a result, the processes described by the danger theory serve as a good inspiration for fault detection systems, especially those with limited resources (cf. [2]). This trend is also visible in the literature review provided in Section 7. The reason is that danger-theory-based approaches can be realized in a highly distributed fashion where the single entities require only simple processing and, thus, are suitable for resource-limited systems. But most importantly, the population-based fault assessment offers smoothing and noise-reduction capabilities that significantly help to lower the false alarm rate, a problem that was prevalent in many previous techniques. Additionally, the basic detection principle of the danger theory, that is, the focus on situations that pose danger to the host, helps to improve the reliability of the system. It concentrates on those faults that endanger the systems’ purpose instead of targeting all possible faults equally.

## 5. Classical AIS Theories and Their Applications

Since the first AIS emerged in the 1990s, much research has been conducted in the field. With growing research interest, the field of AIS became more comprehensive and the areas of application more numerous. Generally, research on AISs can be grouped into three main areas (cf. [90]):

- Immune modeling.
- Theoretical AISs.
- Applied AISs.

Immune modeling is concerned with the biological processes of the HIS and is predominantly covered by immunologists or biologists. Theoretical AISs take inspiration from immune models to develop computational models capable of solving defined problems on a theoretical model. In this context, the mapping from immunological to computational entities remains a problematic task [91]. However, especially applied AISs have gained popularity over the last years as an increasing number of use cases and real-world scenarios arose where AIS can be efficiently applied to solve computational problems [92].

However, over the last two decades, the AIS models have notably evolved. While in the beginning most AISs mimicked adaptive immune response mechanisms only, today more models incorporate processes of both innate and adaptive immunity. For this reason, models including only adaptive immunity are usually referred to as *first generation AIS* and those that include both are denoted as *second generation AIS* [9]. One primary reason for this paradigm shift was the findings on the data fusion capabilities of DCs. Including DCs into an AISs allows the system to correlate data from multiple noisy sensors that help to improve the overall stability of the AIS, especially in the presence of unknown time delays of the signals [9].

AIS have characteristics that make them suitable for optimization or anomaly detection tasks, especially their ability of self-adapting, self-learning, self-organizing, highly parallel processing, and their distributed coordination [7]. Their efficiency can be further improved by auxiliary antigen libraries or concepts from the idiotypic network theory (see Section 5.3). AISs also provide mechanisms for self-regulation by adjusting the lifetime of the cells used and their probability of reproduction [93]. By fine-tuning these parameters, the performance of AIS can be significantly improved [91]. Additionally, these regulatory mechanisms allow the system to adapt to dynamic environments, which in turn is vital as the human body undergoes specific changes over its lifetime [26], which can also be the case for WSNs.

AISs have been shown to perform comparably well on certain benchmark data sets when compared to existing statistical and machine-learning techniques [9]. In some cases, they even presented a more efficient solution than prevalent techniques. Nevertheless, many AIS models have some significant drawbacks that limit their applicability. The most severe ones are their usually high resource consumption (especially for memory) and their ordinarily bad scaling properties [94]. As an example, the authors of [95] compared an AIS-based misbehavior detection with a second instance based on an ANN. They showed that the AIS offers comparable results, in some cases even better than the ANN, but at the cost of resources, especially memory. In their experiments, the AIS-based approach required nearly six times more memory than the ANN approach.

Today, most AIS approaches are derived from one of the following four theories, sometimes called “classical AIS theories” [54]:

- **Negative/positive selection** (mainly based on T cells; see Section 5.1)
- **Clonal selection** (mainly based on B cells; see Section 5.2)
- **Immune network theories** (i.e., idiotypic network theory; see Section 5.3)
- **Danger theory** (i.e., dendritic cell-based algorithms; see Section 5.4)

Aside from these common techniques, several other immunology-inspired algorithms and computational tools have been developed, such as humoral immune response systems [62] and the pattern recognition receptor model [96]. Review work for general AIS approaches is given in [17,54,97,98,99] as well as focused on anomaly detection and IDSs in [13,16,18].

AIS can also be combined with other (learning) techniques to build more efficient ensemble/hybrid systems. One common goal is to decrease the FAR, which is usually high in self-organized (unsupervised) approaches. A typical example are immune genetic algorithms [100,101,102] for optimization problems as well as to lower the FAR of an immunity-based anomaly detection system [103]. A more sophisticated approach was proposed in [104]. This model consists of three evolutionary stages to optimize the overall performance:

1. Gene library evolution [65]
2. Negative selection [105]
3. Clonal selection [106]

For more examples on ensemble/hybrid AIS, we refer to the survey on AIS hybrids presented in [107].

### 5.1. Negative and Positive Selection

In the HIS, negative selection is a process taking place in the bone marrow (for B cells) or the thymus (for T cells). It uses self/non-self discrimination based on a naive model of central tolerance developed in the 1950s [71] and, together with clonal selection, forms the core concepts of the SNS model. The SNS model assumes that the self is defined in early life, and anything that comes later is considered as non-self [25]. The selection process aims at eliminating antibodies (i.e., lymphocytes) that are reactive to entities of the self space. For this purpose, it checks their affinity based on the degree of binding between, for example, T cell receptors and specific antigens. The antibodies failing the selection process are removed from the population.

There are two basic selection processes, namely positive and negative selection. In positive selection, the antibodies are selected to cover the self-space. Thus, only those who match the self are kept while the others are removed. On the other hand, in negative selection, the antibodies are selected to match the non-self space. Nevertheless, positive selection has not been found in the selection of T cells [108]. As a result, the majority of immune-inspired approaches use negative selection. However, which of these two selection processes better suits a given task depends on the size of self and non-self, or their ratio, respectively.

Methods based on negative/positive selection are typically used for classification and pattern recognition problems (e.g., anomaly detection [109]). In anomaly-based IDS, the pathogens represent the potential attacks, and the antibodies are a way to identify that attacks [110].

Inspired by the HIS’ negative selection processes, the negative selection algorithm (NSA) was proposed in 1994 in [105]. The crucial part of the NSA is to find a suitable mapping from the biological entities (e.g., antigens, antibodies, pathogens) to the computational problem. In the area of AISs, the antibodies are usually called detectors as their job is to detect certain circumstances (i.e., the presence of non-self). The detectors are often represented as feature vectors representing antigenic patterns able to detect changes in behavior [111]. Often the problem space is represented by an *n*-dimensional space, and the detectors are hyperspheres that use a matching rule based on an individual membership or distance function (e.g., Euclidean distance). In some NSA-based approaches, immune memory is introduced by promoting detectors that produce many alarms to memory cells with a lower activation threshold [13].

The NSA has two important components: the detectors and the matching rule. The problem of how to generate detectors to minimize their number while maximizing the covering of the non-self space is one of the major fields of research for NSA [112,113]. Usually, the number of detectors required to cover a certain self-space grows exponentially with its size [11]. Also, the shape of the self-space and the detectors has shown to have a significant impact on the number of detectors needed [95].

Related work on the improvement of the detectors focuses on their representation (e.g., binary or real-valued; see [114]), their shape (e.g., hyperspheres or hyperellipsoids; see [115]), the parameters involved in their creation [116], the influence of variable radius [117] as well as the effects of growing or shrinking the detectors surface [118]. An extensive analysis of the effects of different detectors used in NSA as well as the development of improved detector generation algorithms is summarized in [119]. Another way to efficiently cover the entire non-self space is to combine detectors of different types (with their respective matching rules) to reduce the number of holes [11].

Directly intertwined with detectors are the affinity measures (or matching rules) applied. As presented in [54], the metric to measure the affinity (similarity) depends on the choice of vector attributes as it determines the detectors’ shape space type. In [120], different detector shape spaces and suitable affinity metrics are analyzed, such as real-valued shape spaces (with Euclidean distance or Manhattan distance), Hamming shape spaces (with Hamming distance or r-continuous bit rule), and symbolic shape spaces. Also, alternative representations have been proposed such as feature-feature relations [121], or dictionary-based basis decomposition methods [122]. However, choosing an expressive metric is a non-trivial task in most cases.

As stated in [123], most works so far used an antigen representation based on binary feature vectors and applied binary matching rules (e.g., *r*-contiguous matching [105], *r*-chunk matching [114], landscape-affinity matching [124], or Hamming distance matching rules [124,125] and its variations such as Rogers and Tanimoto (R&T) matching rule [124]). Especially the *r*-contiguous matching rule has found application in many NSA-based approaches [11,105,114]. The *r*-contiguous rule matches two strings if they have an identical sequence of *r* bits.

Although approaches based on negative selection had a promising start, they have been found to have severe problems regarding scalability and coverage [14,16]. As pointed out in [126], the required amount of detectors to sufficiently cover the non-self space becomes unmanageable for most problems. The authors of [119] counter this claim and argue that the problem is not with the algorithm itself, but with unsuitable (binary) representations of the problem space (see also [127]).

In addition, there are two common problems with the traditional SNS model applied to AIS, that are a high false positive rate (FPR) when using negative selection (leading to missed anomalies) and a high false negative rate (FNR) when applying positive selection (resulting in a high FAR; cf. [26]). Directly connected with these issues is the problem of a dynamic or changing self as the SNS model assumes a static self that does not change over the lifetime. One way to cope with changing selves is the balance the life cycle of immune cells, enabling an adaptive coverage of the non-self space [65].

Possible solutions to these problems are hybrid approaches. One way to overcome the difficulties with detector coverage is to apply evolutionary algorithms to continuously evolve the detectors, such as GAs [128] or clonal optimization [129]. A prominent example is the evolutionary negative selection algorithm, a hybrid evolutionary immune algorithm that was extended with a niching technique to prevent the algorithm from ending up in a local optima [104]. Also, the usage of gene libraries to avoid random detectors at the initialization is a promising way [13]. These gene libraries lead the generation process of antibodies and can improve the overall efficiency [130].

Another approach dealing with the problem of crisp transitions between the self and non-self space is the combination of negative selection with fuzzy rules [131,132]. Such fuzzy-based NSA have shown favorable characteristics when applied to immunity-based IDS [131]. For a network IDS, also the efficiency of a hybrid AIS combining positive and negative selection has been analyzed [133,134]. Fuzzy rules in combination with Q-learning were used in the cooperative fuzzy artificial immune system proposed in [135] that showed superior properties in comparison with other learning techniques (i.e., C4.5 decision tree, artificial immune recognition system (AIRS), clonal selection algorithm (CLONALG), fuzzy logic controller, and fuzzy Q-learning).

Two of the more complex hybrid approaches are Bayesian artificial immune systems [136,137] and the complex artificial immune system [138]. The former is based on Bayesian networks and is intended for solving hard optimization problems. On the other hand, the complex artificial immune system is a layered model that takes antigens as inputs and proposes antibodies as output. It is best suited for pattern detection problems as it can deal with several transformations such as scaling or rotation of patterns.

However, NSAs have been applied to many problems so far, including anomaly detection [139], fault detection [140], or function optimization [141]. An approach to apply negative selection to an active defense IDS is presented in [142]. Similarly, an immunity-based IDS with a multi-agent architecture is shown in [143]. A survey on NSA applications can be found in [144].

### 5.2. Clonal Selection

Clonal selection theory [28,106] is based on the functions of lymphocytes in immune systems, especially the maturation phase of B cells. The foundation of this theory was introduced by Burnet in 1957 as an explanation for the observed diversity of antibodies during an immune response [27]. The clonal selection theory suggests that lymphocytes activated by antigen-binding trigger a clonal expansion to evolve antibodies with a better affinity to the present antigens. During this clonal expansion, the lymphocytes undergo an affinity maturation where they are subject to somatic hypermutation (a mutation of the cell’s antigen-binding coding sequences) and a subsequent selection mechanism [90]. In hypermutation, the degree of mutation depends on the affinity measure, where a lousy affinity value results in a higher degree of mutation. As a consequence, the generality and coverage of the detection are increased through the process of hypermutation [13].

The clonal selection and the algorithms derived from it, such as the CLONALG [145], are commonly applied to optimization problems and clustering problems (such as pattern recognition) [17]. Additionally, it is often used in conjunction with NSA or an affinity calculator [65]. As the task of affinity evaluation can be partitioned, a parallel version of CLONALG was proposed in [146].

As summarized in [147], the original CLONALG has a relatively high FAR and is not able to cope with dynamic environments. It is impracticable for dense environments making it not suitable for WSN applications. But it can be deployed in a highly distributed manner and offers an efficient detection rate. It allows the development of memory detectors that help to reduce the response time, especially when combined with negative selection. For this reason, an improvement of the original CLONALG was introduced in [148]. Another algorithm based on the CLONALG with influences from artificial immune network (AIN) [149] (see Section 5.3) is the artificial immune recognition system (AIRS) [150,151], one of the first AIS-based supervised learning algorithms. In [146], a version of AIRS is presented in which the affinity evaluation is parallelized.

Although clonal selection approaches rather deal with optimization problems, several attempts of applying it to an anomaly or intrusion detection have been proposed (cf. [152]).

### 5.3. Artificial Immune Networks

Artificial immune networks (AINs) are a class of immune-inspired algorithms that are based on the idiotypic network theory proposed by Jerne [31]. They can be seen as an extension of the clonal selection with the interaction between the antibodies and antigens, or B cells respectively [9]. The AIN model was first proposed in [125] followed by the first AIN algorithm in [153] and an improved version in [154]. Today, one of the most common AIN-based algorithms is aiNet [155] and its variations [156].

Similar to the clonal selection, AIN-based concepts are usually used for optimization and clustering problems as well as data visualization and control where they share properties with ANN [157].

### 5.4. Danger-Theory-Based Approaches

The unique role of APCs and especially the DCs for (innate) immunity is well known since Lafferty and Cunningham’s extended two-signal model from 1975 [33]. DCs are one of the most important immune response regulation mechanisms. Their importance for the immune system became even more evident with the advent of the danger theory in 1994 [35]. Since then, several computational approaches based on the danger theory, or the DCs’ functionality in general have been proposed.

A first in-depth discussion on the potential of the danger theory for AISs was presented in [56]. The authors stressed on the natural anomaly detection capabilities of DCs and their possible applications in computing systems. Thereby, especially a low FPR in combination with a high true positive rate (TPR) are desirable properties for anomaly detection techniques [66]. The anomaly detection is performed by the DCs by correlating the collected antigens with the fused contextual signals. It is necessary to consider the signals in combination as the analysis of particular signals in isolation is insufficient to indicate anomalies [158] or to produce classification [12]. Additionally, the danger theory provides a way of grounding the response by linking it directly to the source for abnormality [56].

danger-theory-based approaches have shown good anomaly detection capabilities while using minimal resources [56]. In contrast to other immune-inspired techniques, the danger theory bases its detection on the presence of danger to the host, represented by so-called danger signals, in combination with an absence of down-regulating safe signals [84]. Thus, danger-theory-based approaches use pre-defined signals to derive the system’s context and react to “dangerous” states rather than all kinds of deviations. These signals are collected over time and in different places allowing the system to leverage spatio-temporal correlation.

Over the years, the danger theory has inspired the development of several AISs. Especially the unique role of the DCs has paved the way for several novel algorithms such as the toll-like receptors (TLR) algorithm [159] and the conserved self pattern recognition algorithm (CSPRA) [96].

The TLR algorithm [159] models the interaction of DC and T cell populations. It uses binary signals (i.e., present and not-present) to stimulate immune responses in a way similar to PAMP signals. For more information on the TLR algorithm and the detailed steps involved, see [160].

Another AIS model influenced by the danger theory is the CSPRA [96]. It allows detecting anomalies by replicating the negative selection of T cells in combination with the self-pattern recognition of APCs. It adds the APCs part of the function as the negative selection is naturally involved from the PRR model.

Nevertheless, the most common danger theory-inspired algorithm is the dendritic cell algorithm (DCA) [12,37,66,76,84,88,110,158,161,162] originally proposed in [37] as part of the so-called “Danger Project” [56]. The DCA is suitable for use in resource-constrained systems and can perform context-aware anomaly detection. Both are properties desirable for fault detection approaches in WSNs. For this reason, an introduction to the DCA, its working principle, and its further developments are presented in Section 6.

## 6. The Dendritic Cell Algorithm

The dendritic cell algorithm (DCA) was one of the first algorithms that used the functioning of dendritic cells as suggested by the danger theory for solving computational problems. Its initial version (also called “classical DCA”) was introduced by Julie Greensmith in 2005 [37]. The DCA is based on the DCs’s ability to combine multiple signals to assess the current context of their environment. In contrast to other AISs, it relies on the correlation of information from the population of DCs rather than pattern-matching based on similarity metrics [66]. Further differences to other AIS algorithms are the combination of multiple signals from diverse sources, as well as the correlation of signals with antigens in a temporal and distributed manner to form a context-aware anomaly detection system [12].

To confirm the algorithm’s basic working principle, it was initially used to classify data provided by the UCI Wisconsin breast cancer data set with signals derived from the data attributes. The original intention for the development of the DCA was its use in an immune-inspired IDS where it has then been applied for the detection of port scans and the detection of botnets in computer networks (cf. [76]), as well as for attack detection in an Open Platform Communications Unified Architecture (OPC UA) framework [163].

### 6.1. Working Principle

**Figure 4:**
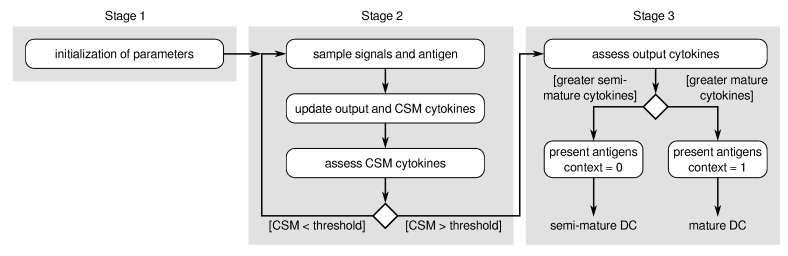
Key features of DC biology used in the DCA (after [84], Figure 5.3).

The DCA describes an abstract model of the functioning of dendritic cells based on Matzinger’s danger theory [25]. For this purpose, it uses a population of abstracted dendritic cells, each with a collection of antigens the cell encountered during its life, a finite lifetime with a pre-defined threshold, and a contextual value depending on the concentration of the input signals as described below. As depicted in Figure 4, the original DCA consists of three main stages [12]:

1. Initialization (setting of various parameters);
2. Cell update (event-driven update of variables);
3. Data aggregation.

#### 6.1.1. Cell Update

Until the lifetime of a cell is exceeded (i.e., update stage), each cell iteratively performs three functions:

1. The sampling of antigens;
2. The update of the input signals;
3. The calculation of the cell’s interim output signals.

The core mechanism of the cell update stage is the collection of antigens and signals over the DCs’ lifetime. Four types of input signals are combined to acquire contextual information on the status of the target system (cf. [37]). They are analogous to the natural signals observed in the HIS [35]:

- PAMP (*P*) — signals that are known to be pathogenic.
- Safe (*S*) — signals that are known to be normal.
- Danger (*D*) — signals that indicate changes in behavior.
- Inflammatory (*I*) — signals that amplify the other signals.

Based on these input signals, the DCA calculates three intermediate output values:

- co-stimulatory molecule (CSM): expresses the cell’s maturation status.
- Semi-mature value: response to a safe environment.
- Mature value: response to a dangerous environment.

**Figure 5:**
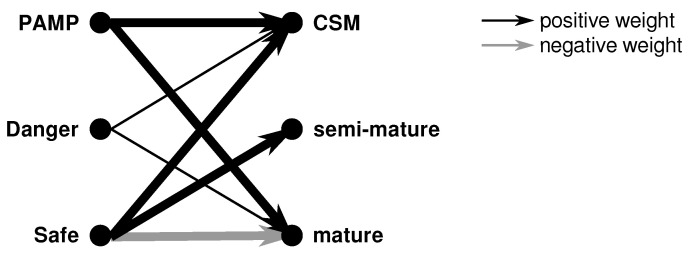
Abstract model of the DCA signal processing (after [84], Figure 5.4).

The correlation of input-to-output signals is shown in Figure 5. In this illustration, the thickness of the lines expresses the transforming weights.

In biology, PAMP are occurrences known to be not produced by the host, hence, a clear sign of danger [84]. In the DCA, they lead to an increase in CSM and mature output signals resulting in an earlier maturation with an anomalous context (i.e., mDC). The CSM expresses the maturation status of the cell, that is, whether the cell is ready for antigen presentation [84]. Danger signals are indicators of possible anomalies and influence the CSM and mature output signals but, as can be seen in Figure 5, much lower than the PAMP signals [84]. On the other hand, safe signals suppress the production of the mature output signal (negative weight) and cause an increase in the semi-mature output value. Still, they contribute to the DC’s maturation (i.e., increase of the CSM value).

The intermediate output signals are derived from the input according to the equations presented in [37]:

(1) $$\begin{array}{cc} C_{csm} & =\left(\underline{2}\sum_{i=0}^{I}P_{i}+\underline{1}\sum_{i=0}^{I}D_{i}+\underline{2}\sum_{i=0}^{I}S_{i}\right)\cdot \left(1+IC\right) \end{array}$$

(2) $$\begin{array}{cc} C_{semi-mature} & =\left(\underline{0}\sum_{i=0}^{I}P_{i}+\underline{0}\sum_{i=0}^{I}D_{i}+\underline{3}\sum_{i=0}^{I}S_{i}\right)\cdot \left(1+IC\right) \end{array}$$

(3) $$\begin{array}{cc} C_{mature} & =\left(\underline{2}\sum_{i=0}^{I}P_{i}+\underline{1}\sum_{i=0}^{I}D_{i}+\left(\underline{-3}\right)\sum_{i=0}^{I}S_{i}\right)\cdot \left(1+IC\right) \end{array}$$

where $C_{csm}$, $C_{semi-mature}$, and $C_{mature}$ are the intermediate output signals respectively, $P_{i}$ represent the PAMP signals, $D_{i}$ represent the danger signals, $S_{i}$ represent the safe signals, and IC are inflammatory cytokines. The respective weights of the single terms (underlined numbers) are based on the suggestions in [158].

One effect present in this equation, but not shown in Figure 5, is inflammatory cytokines (IC) expressing an already ongoing infection. These signals have an amplifying effect on the other three input signals (i.e., PAMP, danger, and safe).

#### 6.1.2. Data Aggregation

As shown in Figure 4, when a dendritic cell reaches the end of its life (i.e., its CSM value exceeds a defined threshold), its interim output signal concentrations are assessed to define its contextual status (i.e., semi-mature or mature). Based on this information, the accumulated antigens are classified based on whether more dendritic cells experienced this antigen in a normal or an anomalous context (i.e., binary classification). In the case of a dominating semi-mature signal, the group of antigens is assigned a “normal” context; otherwise, it is assigned an “anomalous” context. As opposed to most other immune-inspired algorithms, the DCA uses the collected antigens merely for labeling and tracking of data rather than for detection purposes.

#### 6.1.3. Algorithmic Properties

The DCA was initially designed as an offline anomaly detection algorithm to be applied to network intrusion detection. Due to the replication of the DCs’ functioning, it shows similarities with certain filtering techniques. In addition, the DCA has lower computational complexity (in comparison with other machine-learning techniques) and it does not require extensive training periods [76]. For this reason, it has also shown preliminary success in resource-constrained applications, such as sensor networks and mobile robotics [84].

Since the lifespan of the individual DC instances are limited and influenced by the environment, the DCA forms a filter-based correlation algorithm that includes a time window effect that reduces false positive errors [88]. Initial experiments on the DCA have shown a high accuracy [66], but, depending on the application, also a comparably high FAR (cf. [135]). Additionally, the initial DCA does not involve any learning mechanisms regarding the selection, mapping, and weighting of the signals used, making manual tuning and preparation necessary [162]. Therefore, there is great potential for future improvements regarding the signal sources and their respective mapping.

### 6.2. Variants and Further Developments

The classical DCA gained promising results but contained stochastic elements and required the fine-tuning of more than ten parameters that made it more challenging to apply. Consequently, its foundation was theoretically analyzed and some simplifications were introduced based on which the deterministic dendritic cell algorithm (dDCA) was proposed in [76]. The main changes concerned:

- The lifetime of the dendritic cells.
- The way antigens are sampled and stored.
- The processing of the input signals.

Regarding the latter, the calculation of the interim output signals was significantly reduced to one signal expressing the maturation (lifetime) status of the cell (i.e., co-stimulatory signal) and a second one keeping track of the experienced system context (i.e., context value). In the dDCA, the co-stimulatory signal is calculated with

(4) csm=S+D

and the context value is expressed as

(5) k=D−2S

where *D* refers to the sum of danger signals and *S* to the sum of safe signals, respectively. The theoretical analysis for the reduction in these two interim signals is provided in [164]. However, for both, only the danger and safe signals are used. Thus, the special roles of the PAMP and inflammatory processes were neglected. As a consequence, the parameters of the dDCA were reduced to:

- The input signals (danger and safe).
- The dendritic cell population size.
- The lifetime of the single cells.

While the input signals determine the detection capabilities of the dDCA, the population size and lifetime of the dendritic cells influence the smoothing and noise reduction properties of the algorithm, both responsible for decreasing the false positives rate (cf. [162]).

The classical DCA and the dDCA were used for a (binary) classification of offline data. Therefore, all data must be already available when the algorithm is applied. However, many anomaly detection systems require runtime (or even real-time) detection capabilities. A first approach to transform the DCA into a runtime detection algorithm by utilizing segmentation techniques is discussed in [165]. To avoid the need for segmentation, the authors of [166] proposed the minimized dDCA (min-dDCA). Their min-dDCA replaced the usual population sampling strategy with a one-to-one correlation between signals and antigens. Most importantly, they reduced the population size to one single dendritic cell with a lifetime of one iteration. Thus, the dendritic cell assigns a context to the present antigen in each iteration. But the runtime processing comes at the cost of missing result smoothing and noise reduction.

## 7. Literature Review on Immune-Inspired Approaches

In this section, we provide a review of immune-inspired methods for fault detection. The search was conducted by searching the publication databases *IEEE Xplore*, *ACM Digital Library*, *ScienceDirect*, *Springer Link*, and *Web of Science* for the years 2002–2022 using the search string (items are AND connected):

- “AIS” OR “immune” OR “immunity”
- “anomaly” OR “abnormal” OR “fault”
- “wireless sensor network” OR “WSN” OR “sensor node”

To give our review a broader scope, we also included related works that target computer networks, IoT applications, cyber-physical systems (CPSs), and ad hoc networks. The found papers were manually filtered by reviewing the titles and abstracts followed by manual inspection of the content of the remaining papers. A summary of the key characteristics of the final set of relevant papers is provided in Table 1. For an extensive summary of AIS applications for connected devices in general (i.e., IoT devices), we refer an interested reader to the systematic review provided in [167].

The majority of AIS research was focused either on negative selection or the danger theory [13,90,168]. Especially in the field of WSNs, there is a noticeable trend towards danger-theory-based approaches. The reason is mainly scaling problems of the NSA that are even worse when being applied to real network traffic [126]. Secondly, the danger theory’s distributed and simple concept is suitable for most WSN applications [147]. Therefore, we will give a brief overview of the application of immune-inspired techniques for computational problems in general and specific to their use in WSNs.

### 7.1. Anomaly Detection

Based on the aim of the HIS to keep the host healthy by eliminating threats to proper functioning, several researchers claimed that the HIS forms a natural anomaly detection system [13,16,17]. It can detect pathogens without prior knowledge of their structure [16] and offers a very low FPR, as well as FNR [13], thus making it a perfect example of a (distributed) anomaly detection system.

A combination of negative and clonal selection for network anomaly detection in WSNs is proposed in [91] and an extension of it in [15]. The authors define antigens as random low-level bit patterns and, as in the HIS, let the immunity-inspired mechanisms take care of their evolution.

The ability of the immune system to cope with a dynamic environment is leveraged in [169]. In this work, the authors developed an anomaly detection approach inspired by the clonal selection found in the HIS. Aside from a comparably high accuracy, their approach was shown to be able to cope with a slowly changing environment without triggering false alarms.

In general, AIS-inspired anomaly detection found applications in a great number of different fields [17], such as virus detection [170], intelligent spam mail filter [171], credit card fraud detection [172] or different other computer-security-related topics [18,124].

**Table 1:** Overview of Immune-inspired Approaches.

		Scope			Locus		Immune Concept				Target System					
Authors	Year	Data Anomaly	Network Intrusion	Fault Diagnosis	Centralized	Distributed	Negative Selection	Clonal Selection	Immune Network	Danger Theory	Sensor Networks	Computer Networks	Other	Adaptability	Learning	Notes
Harmer et al. [124]	2002	○	●	○	○	●	●	○	○	○	●	○	○	●	◐	
Sarafijanović and Le Boudec [15]	2005	○	●	○	○	●	●	●	○	○	○	●	○	◐	◐	Initial concept confirmed by simulations
Boukerche et al. [173]	2007	○	●	○	●	○	●	○	○	○	○	●	○	◐	◐	
Drozda et al. [174]	2007	○	●	○	●	○	●	○	○	○	●	○	○	●	◐	Apply random-generate-and-test process
Powers and He [175]	2008	○	●	○	●	○	●	○	○	○	○	●	○	●	●	Negative selection with GA
Liu et al. [176]	2008	○	●	○	○	●	●	●	○	○	●	○	○	●	◐	Concept simulated with TOSSIM
Yang et al. [177]	2010	○	○	●	○	●	●	●	○	○	○	●	○	◐	◐	
Greensmith et al. [66]	2010	○	●	○	●	○	○	○	○	●	○	●	○	●	○	Summary of seminal works on the DCA
Bo Chen [111]	2010	○	○	●	○	●	●	●	○	○	●	○	○	◐	◐	Applied to structural health monitoring
Laurentys et al. [178]	2011	○	○	●	●	○	●	●	○	○	○	○	●	◐	◐	
Ou et al. [168]	2013	○	●	○	○	●	○	○	○	●	○	●	○	●	○	Utilizes an adapted DCA
Shamshirband et al. [135]	2014	○	●	○	●	○	○	○	○	●	●	○	○	◐	●	
Salvato et al. [169]	2015	○	○	●	●	○	●	●	○	○	●	○	○	●	◐	
Xiao et al. [179]	2015	●	○	●	○	●	○	○	○	●	●	○	○	●	○	
Rizwan et al. [180]	2015	○	●	○	○	●	●	○	○	○	●	○	○	○	○	Applies a form of artificial vaccination
Cui et al. [181]	2015	○	○	●	●	○	○	○	○	●	●	○	○	●	○	DCA-based fault diagnosis
Mohapatra and Khilar [182]	2017	○	○	●	●	○	●	●	○	○	●	○	○	◐	◐	
Sun et al. [183]	2018	○	●	○	○	●	●	○	○	○	●	○	○	○	○	Offline & computation-intense training
Alaparthy and Morgera [73]	2018	○	●	○	●	○	○	○	○	●	●	○	○	●	○	
Li and Cai [184]	2018	◐	○	●	●	○	○	○	○	●	●	○	○	●	○	
Alizadeh et al. [185]	2018	○	○	●	●	○	○	○	○	●	●	○	○	●	○	DCA-based fault diagnosis
Akram and Raza [186]	2018	○	○	●	●	○	○	○	○	●	○	○	●	●	○	DCA-based fault diagnosis
Aldhaheri et al. [187]	2020	○	●	○	●	○	○	○	○	●	○	○	●	●	●	DCA-based IDS
Bejoy et al. [188]	2022	○	●	○	●	○	●	●	○	○	○	●	○	●	●	

● considered; ◐ partly considered; ○ not considered.

### 7.2. Intrusion Detection Systems (IDS)

The application of AIS for network anomaly detection, as part of an IDS has drawn much of the attention of the research community [54,105,173,189,190]. In this context, the expected behavior is usually considered as the self space, and any deviation from it counts as non-self [191]. To increase efficiency while reducing the FAR, hybrid approaches can be beneficial (cf. [175]).

The majority of previous works on immune-inspired IDSs target computer networks [192,193], IoT devices [187], or CPSs [188]. In additions, several works apply immune principles to IDSs applicable to WSNs, such as the negative selection-based IDS for WSNs named WSN-NSA [183], the multi-level IDS for WSNs [73], or Co-FAIS [135], a danger-theory-based IDS that utilizes fuzzified network traffic in WSNs. While early works primarily use negative selection as their underlying detection strategy, a shift towards danger-theory-based concepts is noticeable as discussed above.

For applying AISs to WSNs, the mapping of entities of immunity to those of the WSN is an especially crucial task. For network-based approaches, often the antigen is derived from information extracted from network packets and stored in feature vectors [168]. On the other hand, host-based systems often use operating system (OS)-related information, such as system calls, to derive the antigens [66]. Examples of immune-inspired IDS applied to WSNs are given in [174,176].

### 7.3. Fault Detection

Concerning the use of AISs for fault detection, immune-inspired approaches can also be used to detect internal deviations rather than focusing on attacks from the outside (similar to the HIS). In this context, several fault diagnosis systems inspired by the HIS have been proposed [194]. As with IDSs, also such systems often assume a fault-free system behavior at the early stages [195]. However, many of these approaches suffer from a high FPR [178].

Based on immune models, a maintenance architecture able to detect faulty behavior has been proposed in [196]. Another network fault diagnosis approach based on AIS is presented in [177]. Specialized systems to detect hardware faults are introduced in [177], as well as systems leveraging co-stimulation in [5,197]. In [184], a danger-theory-based fault diagnosis is applied to identify abnormalities in the energy consumption patterns of monitored equipment in a CPS.

Targeting WSN, a danger-theory-based data-cleaning concept for environmental monitoring WSNs is presented in [179], which considers missing data, faults, and systematic errors in the provided sensor measurements. The fault detection technique in [198] is tailored for WSNs, and consists of a linear-vector-quantization-based training phase and a subsequent AIS-based diagnosis mode. The fault diagnosis algorithm proposed in [182] uses a clonal selection-inspired approach to identify hard and soft faulty sensor nodes in a WSNs. Additionally, for structural health monitoring (SHM) with WSNs, some immune-inspired approaches have been proposed [111,199].

However, as argued in [94], an efficient fault detection system could combine AIS with an artificial endocrine system. The AIS is suitable for detecting low-level faults that can be corrected locally, and the artificial endocrine system is better suited to recognize chronic faults.

An overview of detection approaches based on negative selection, clonal selection, and immune networks is available in [180,200]. For a general overview of fault detection strategies and approaches, we refer to the survey on fault detection in WSNs given in [22,23].

### 7.4. DCA-Based Fault Detection

In the following, we provide an overview of DCA-based fault detection approaches, which extends the review of DCA-based methods presented in [201].

One of the first works that used the DCA for fault detection was presented in [181]. The authors applied the principles of the DCA on a fault diagnosis system for rotating machinery in industrial facilities. Their input signals focused on the vibration pattern acquired from vibration sensors. Five signals derived from the vibration data, such as kurtosis, were considered. The authors claimed that their approach achieved an overall diagnostic accuracy of over 93 %. However, they gave no details on their implementation and signal combination.

In [185], a DCA-based fault detection system for sensor faults in wind turbines is proposed. The approach used redundant sensor measurements to acquire the input signals for the DCA-based fault detection. In addition, the authors compared their approach with a NSA-based implementation. The results show that both immune-inspired techniques offer a similarly good fault detection rate, but the NSA suffered from a higher false alarm rate.

So far, the only work that incorporates node-level information in an immune-inspired fault detection approach is presented in [186], which was applied to a robotic system. The authors defined a set of so-called health indicators that are used as input for the DCA. These health indicators are derived from operational characteristics on the node level, such as energy consumption, battery level, component temperature readings, and task completion status. All proposed health indicators are calculated as the difference between two consecutive measurements. The authors present an extensive analysis of their approach that resulted in an overall fault detection rate of 98% with only 0.128% false alarm rate.

## 8. Open Problems and Research Directions

Most AISs and immune-inspired approaches are derived from one of the four “classical AIS theories”. Concerning anomaly or fault detection, primary approaches based on negative selection (self/non-self discrimination) or techniques based on the functioning of the dendritic cells (contextual information fusion) have been proposed. While negative selection techniques dominated the early stages of AIS-based detection systems, an increasing number of dendritic-cell-based algorithms have been proposed over the years (cf. Table 1). The reason for this is the usually high memory consumption and comparably high false positives rate of most negative selection approaches. Both disqualify negative selection approaches, especially from a meaningful use in resource-constrained systems such as WSNs [147].

Aside from the resource requirements, there are several challenges and open problems concerning the use of immune mechanisms for fault detection in WSNs. In the following, we will discuss these issues and the corresponding future research directions toward effective and efficient sensor node fault detection.

### 8.1. Entity Mapping

One of the most challenging tasks in the adoption and adaptation of immune principles to solve computational problems is a suitable mapping of the biological entities to computational counterparts. In this context, several researchers tried to recreate an AIS including entities similar to those found in the HIS. For example, the DCA uses a population of abstract dendritic cells. Similarly, the IDS presented in [202] uses a virtual thymus that is inspired by its biological similitude. Some researchers even argue that the basic structure of WSNs shows a certain similarity with the biological entities involved in human immunity.

However, adequate mapping is not always easy to find or is even close to impossible. Additionally, the general characteristics of the considered biological systems need to be considered when developing computational models of certain immune mechanisms. As discussed in Section 3.1 and Section 3.3, the HIS involves a vast amount of different cells to distributively perform their tasks. Consequently, in the HIS, quantity has often more effect on the process’ performance than quality.

In computing systems, it is usually the other way around. Most technical systems simply do not have enough components to even remotely replicate the processes of the biological immune system. Although there are WSN that incorporate thousands of sensor nodes, these numbers are no comparison to the number of cells cooperatively proving immunity. Consequently, researchers have to develop suitable abstractions of the underlying processes or need to come up with creative solutions to overcome the limitations of computing systems.

### 8.2. Feature Selection

In most immune-inspired approaches, the definition and selection of the used features is a manual process that requires a certain level of knowledge and expertise of the target system. This is especially true for the input signals of DCA-based approaches (e.g., danger and safe). Several authors incorporated dimensionality reduction techniques, such as the principal component analysis (PCA) or even mechanisms based on self-organizing maps (SOMs), to introduce some automatism in their feature selection process. However, even in the most sophisticated of these approaches, human intervention, or at least supervision, is necessary to ensure good results.

Additionally, the majority of immunity-based fault detection systems derive the input features purely from the sensor data. In this context, most fault models assume that faults significantly alter the sensed data. However, such data analytical detection approaches suffer from a disability in distinguishing rare but proper events from data anomalies caused by soft faults (cf. [3], Section 2.4). The inclusion of node-level diagnostic information is only sparsely addressed in related work (cf. [23,203]).

In addition, the majority of related works focus on the suitable adoption and adaptation of immune mechanisms to develop effective detection algorithms. The algorithms’ input data are mostly treated as a means to an end but are often not particularly considered. However, we found that often the input data are the essential part, and the detection algorithm is mostly a vehicle for the automated assessment. In other words, the best and most efficient algorithm still relies on the quality of the input data. Nevertheless, the algorithm has an impact on the characteristics of the detection approach and, thus, its final results regarding its effectiveness and efficiency.

### 8.3. Learning Capabilities

While negative and clonal selection-based techniques offer at least some degree of learning, especially approaches inspired by the danger theory, such as those based on the DCA, do not include learning capabilities. However, several works proposed concepts to incorporate machine learning for two purposes: (i) an automated selection, mapping, and weighting of the input parameter and/or (ii) an introduction of immune memory. The latter refers to the capability of the system to learn from previous fault encounters to achieve a faster reaction in case the same situation is experienced again.

Concerning approaches based on the DCA, a theoretical analysis of the algorithmic basis revealed that it is a collection of linear classifiers [164]. To cope with these limitations, several works suggested replacing the classification stage of the DCA with machine-learning capabilities (cf. [201]). Especially the use of fuzzy inference systems has gained promising results [204]. Such approaches, however, entail a significant overhead on the memory and processing that prevent them from being used in resource-constrained systems such as WSNs.

As a consequence, resource-efficient ways need to be found to incorporate learning capabilities into lightweight approaches suitable for resource-constrained sensor nodes. Some seminal works have successfully combined immune mechanisms with other bio-inspired techniques, such as GA, to form an efficient hybrid system that is also capable of learning (cf. [175]).

### 8.4. Data Correlation

The majority of found approaches utilize the temporal correlation of subsequent measurements of the sensor nodes for their fault detection. Since the HIS also performs a spatial correlation of the information collected in the tissue, the detection strategy of respective sensor node fault detection approaches could also support spatio-temporal correlations. For example, the detection could consider the data of several sensor nodes within a certain neighborhood. Such a spatio-temporal fault detection, however, requires a suitable format of the input data that is not trivial to define.

## 9. Conclusions

In this article, we presented a literature review of immune-inspired fault detection approaches for wireless sensor networks (WSNs). After a brief introduction and the motivation for applying immune-inspired techniques to detect sensor node faults in Section 1, we provided an excursion to the history of immunology and the prevalent immunological models in Section 2. In this context, we highlighted the unique properties of the immune system that are desirable for computational detection approaches as discussed in Section 3. Considering their use for fault detection, especially immune mechanisms as described by the danger theory (discussed in Section 4), show promising characteristics. In Section 5, the four “classical” AIS theories are presented. For fault detection purposes, especially the danger-theory-based dendritic cell algorithm (DCA), as discussed in Section 6, showed promising results. As the core of this article, we discussed related works proposed in the last two decades in Section 7. We combined the found information with findings from our previous works to elaborate on the most important limitations and shortcomings of current immune-inspired fault detection approaches based on which we provide corresponding future research directions in Section 8.

Regarding the limitations of current approaches, we particularly found that the detection of sensor node faults has most often been considered a data anomaly detection task and was often purely performed on the sensor data only. Utilizing data anomaly detection for fault diagnosis suffers from a crucial problem: anomalies do not need to be caused by faulty sensor nodes. Similarly, not all node faults cause distinct irregularities in the reported sensor data. Therefore, such approaches suffer from a disability in distinguishing between data events and fault-induced deviations.

Aside from the input data, there are some additional open questions concerning an appropriate mapping between biological and computational entities. Additionally, a suitable selection scheme for the most expressive features utilizable for fault detection remains a non-trivial task. Moreover, the majority of current immune-inspired detection approaches have limited to no learning capabilities, which hinders the exploitation of effective concepts such as immune memory found in human immunity. Similarly, most approaches focus on temporal correlations in the data and neglect spatial information that together could be leveraged for an effective spatio-temporal fault detection scheme.

To sum up, a lot of effort has been spent in the last two decades for the development of sensor node fault detection approaches that took inspiration from processes found in the human immune system (HIS). Although they yielded comparably good and promising results, there are still many unresolved issues and open questions that need to be answered to achieve effective yet efficient fault detection in WSN.

## Abbreviations

The following abbreviations are used in this manuscript:

AIN	artificial immune network
AIRS	artificial immune recognition system
AIS	artificial immune system
ANN	artificial neural network
APC	antigen-presenting cell
CLONALG	clonal selection algorithm
CPS	cyber-physical system
CSM	co-stimulatory molecule
CSPRA	conserved self pattern recognition algorithm
DC	dendritic cell
DCA	dendritic cell algorithm
dDCA	deterministic dendritic cell algorithm
FAR	false alarm rate
FNR	false negative rate
FPR	false positive rate
GA	genetic algorithm
HIS	human immune system
IDS	intrusion detection system
INS	infectious non-self
IoT	Internet of Things
min-dDCA	minimized dDCA
NSA	negative selection algorithm
OPC UA	Open Platform Communications Unified Architecture
OS	operating system
PAMP	pathogen associated molecular patterns
PCA	principal component analysis
PRR	pattern recognition receptor
SHM	structural health monitoring
SOM	self-organizing map
SNS	self/non-self
TLR	toll-like receptors
TNR	true negative rate
TPR	true positive rate
WSN	wireless sensor network
